# Elucidation of the critical epitope of an anti-EGFR monoclonal antibody EMab-134

**DOI:** 10.1016/j.bbrep.2018.03.010

**Published:** 2018-04-12

**Authors:** Mika K. Kaneko, Shinji Yamada, Shunsuke Itai, Yao-Wen Chang, Takuro Nakamura, Miyuki Yanaka, Yukinari Kato

**Affiliations:** a Department of Antibody Drug Development, Tohoku University Graduate School of Medicine, 2-1 Seiryo-machi, Aoba-ku, Sendai, Miyagi 980-8575, Japan; b New Industry Creation Hatchery Center, Tohoku University, 2-1, Seiryo-machi, Aoba-ku, Sendai, Miyagi 980-8575, Japan

**Keywords:** mAb, monoclonal antibody, SCC, squamous cell carcinoma, DMEM, Dulbecco's Modified Eagle's Medium, EDTA, ethylenediaminetetraacetic acid, BSA, bovine serum albumin, PBS, phosphate-buffered saline, FBS, fetal bovine serum, DAB, 3,3-diaminobenzidine tetrahydrochloride, EGFR, Epitope mapping, Monoclonal antibody, Oral cancer

## Abstract

The epidermal growth factor receptor (EGFR) is a type-1 transmembrane receptor tyrosine kinase, which activates the downstream signaling cascades in many tumors, such as oral and lung cancers. We previously developed EMab-134, a novel anti-EGFR monoclonal antibody (mAb), which reacts with endogenous EGFR-expressing cancer cell lines and normal cells independent of glycosylation in Western blotting, flow cytometry, and immunohistochemical analysis. EMab-134 showed very high sensitivity (94.7%) to oral squamous cell carcinomas in immunohistochemical analysis. In this study, we performed enzyme-linked immunosorbent assay (ELISA), flow cytometry, and immunohistochemical analysis to determine the epitope of EMab-134. A blocking peptide (375–394 amino acids of EGFR) neutralized the EMab-134 reaction against oral cancer cells in flow cytometry and immunohistochemistry. The minimum epitope of EMab-134 was found to be the _377-_RGDSFTHTPP_−386_ sequence. Our findings can be applied for the production of more functional anti-EGFR mAbs that in turn can be used for antitumor treatments.

## Highlights

• We previously produced EMab-134, a novel sensitive and specific anti-EGFR mAb.
• EMab-134 is useful in Western blot, flow cytometry, and IHC analyses.
• A blocking peptide neutralized EMab-134 reaction against oral cancer cells.
• The minimum epitope of EMab-134 was found to be _375-_RGDSFTHTPP_−384_ sequence.

## 1 Introduction

Epidermal growth factor receptor (EGFR) is a type-1 transmembrane glycoprotein, which is involved in cell growth and differentiation [1]. EGFR belongs to the human EGFR (HER) family of receptor tyrosine kinases [2], [3], [4] and forms homo- or heterodimers with other members of the HER family, such as HER2 [5] and HER3 [6]. EGFR overexpression is observed in many cancer types, including head and neck, lung, colorectal, breast, pancreatic, kidney, ovary, bladder, and prostate cancers [7].

Monoclonal antibodies (mAbs) against EGFR have been developed for cancer treatment; e.g., cetuximab (a mouse–human chimeric mAb; IgG_1_) against head and neck and colorectal cancers; panitumumab (a fully human mAb; IgG_2_) against colorectal cancers; and necitumumab (a fully human mAb; IgG_1_) against non-small cell lung cancers [8], [9], [10]. Anti-EGFR mAbs possess diverse functional mechanisms, such as blocking ligand binding, blocking dimerization, EGFR endocytosis, antibody-dependent cellular cytotoxicity, and complement-dependent cytotoxicity.

In our previous study, we immunized mice with EGFR-expressed glioblastoma cells or purified recombinant EGFR to produce EMab-134 clone (IgG_1_, kappa), which reacted with endogenous EGFR of oral cancers in flow cytometry, Western blotting, and immunohistochemistry [11]. In immunohistochemical analysis, EMab-134 stained 36 of 38 (94.7%) oral cancer specimens.

In this study, we evaluated the binding epitope of EMab-134 using enzyme-linked immunosorbent assay (ELISA), flow cytometry, and immunohistochemistry.

## 2 Materials and methods

### 2.1 Cell lines

LN229/EGFR was previously established [11], [12]. HSC-3 (oral squamous carcinoma cell line from tongue) was obtained from the Japanese Collection of Research Bioresources Cell Bank (Osaka, Japan). LN229/EGFR and HSC-3 were cultured in Dulbecco's Modified Eagle's Medium (DMEM; Nacalai Tesque, Inc., Kyoto, Japan), supplemented with 10% heat-inactivated fetal bovine serum (Thermo Fisher Scientific Inc., Waltham, MA), 100 units/ml penicillin, 100 μg/ml streptomycin, and 25 μg/ml amphotericin B (Nacalai Tesque, Inc.), and incubated at 37 °C in a humidified atmosphere of 5% CO_2_ and 95% air.

### 2.2 Enzyme-linked immunosorbent assay (ELISA)

Synthesized EGFR (Accession No.: NP_005219) peptides using PEPScreen (Sigma-Aldrich Corp., St. Louis, MO) and extracellular domain of EGFR (EGFRec) were immobilized on Nunc Maxisorp 96-well immunoplates (Thermo Fisher Scientific Inc.) at 10 μg/ml for 30 min at 37 °C or over night at 4 °C. After blocking with SuperBlock T20 (PBS) Blocking Buffer (Thermo Fisher Scientific Inc.), the plates were incubated with purified EMab-134 (10 μg/ml), followed by a 1:2000 dilution of peroxidase-conjugated anti-mouse IgG (Agilent Technologies Inc., Santa Clara, CA). The enzymatic reaction was conducted using 1-Step Ultra TMB-ELISA (Thermo Fisher Scientific Inc.). Optical density was measured at 655 nm using an iMark microplate reader (Bio-Rad Laboratories, Inc., Berkeley, CA). These reactions were performed at 37 °C with a total sample volume of 50–100 μl.

### 2.3 Flow cytometry

Cells were harvested after brief exposure to 0.25% trypsin/1 mM ethylenediaminetetraacetic acid (EDTA; Nacalai Tesque, Inc.). After washing with 0.1% bovine serum albumin in PBS, the cells were treated with EMab-134 (10 μg/ml) or EMab-134 (10 μg/ml) plus peptides (10 μg/ml) for 30 min at 4 °C, followed by treatment with Alexa Fluor 488-conjugated anti-mouse IgG (1:1000; Cell Signaling Technology, Inc., Danvers, MA). Fluorescence data were acquired using the Cell Analyzer SA3800 (Sony Corp., Tokyo, Japan).

### 2.4 Immunohistochemical analyses

This study examined one patient with oral cancer who underwent surgery at Tokyo Medical and Dental University [11]. The Tokyo Medical and Dental University Institutional Review Board reviewed and approved the use of human cancer tissues. Written informed consent was obtained for the use of human cancer tissue samples. Histological Sections (4-μm thick) were directly autoclaved in EnVision FLEX Target Retrieval Solution High pH (Agilent Technologies Inc.) for 20 min. After blocking with SuperBlock T20 (PBS) Blocking Buffer (Thermo Fisher Scientific Inc.), sections were incubated with EMab-134 (5 μg/ml) or EMab-134 (5 μg/ml) plus peptides (5 μg/ml) for 1 h at room temperature, treated using an Envision+ kit (Agilent Technologies Inc.) for 30 min. Color was developed using 3,3-diaminobenzidine tetrahydrochloride (DAB; Agilent Technologies Inc.) for 2 min, and counterstained with hematoxylin (FUJIFILM Wako Pure Chemical Industries Ltd., Osaka, Japan).

### 2.5 Determination of binding-affinity by ELISA

EGFRec and a 375–394-amino acid (aa) peptide were immobilized at 1 μg/ml and 10 μg/ml, respectively. The plates were incubated with serially diluted antibodies (5 pg/ml – 50 μg/ml) followed by 1:2000 diluted peroxidase-conjugated anti-mouse IgG (Agilent Technologies Inc.). The dissociation constants (*K*_D_) were obtained by fitting the binding isotherms using the built-in one-site binding models in Prism software.

## 3 Results and discussion

We previously developed EMab-134, a novel anti-EGFR mAb, which exhibited high specificity and sensitivity against human EGFR [11]. EMab-134 was found to be useful not only for flow cytometry and Western blotting but also for immunohistochemical analyses with paraffin-embedded tissues. Therefore, the determination of the binding epitope of EMab-134 was deemed critical for developing a molecular targeted therapy against EGFR.

In this study, three deletion mutants of EGFR were expressed transiently in CHO-K1 cells, including dN152 [corresponding to 152–1210 aa], dN313 (corresponding to 313–1210 aa), and dN482 (corresponding to 482–1210 aa) (data not shown). All deletion mutants of EGFR contained an N-terminal PA tag [13] and were analyzed using flow cytometry for EMab-134 epitope mapping. NZ-1, an anti-PA tag mAb, detected all deletion mutants of EGFR. In contrast, EMab-134 did not react with dN482 (data not shown). These results indicated that the N-terminus of EMab-134 epitope existed between aa 313 and 482.

**Fig. 1:**
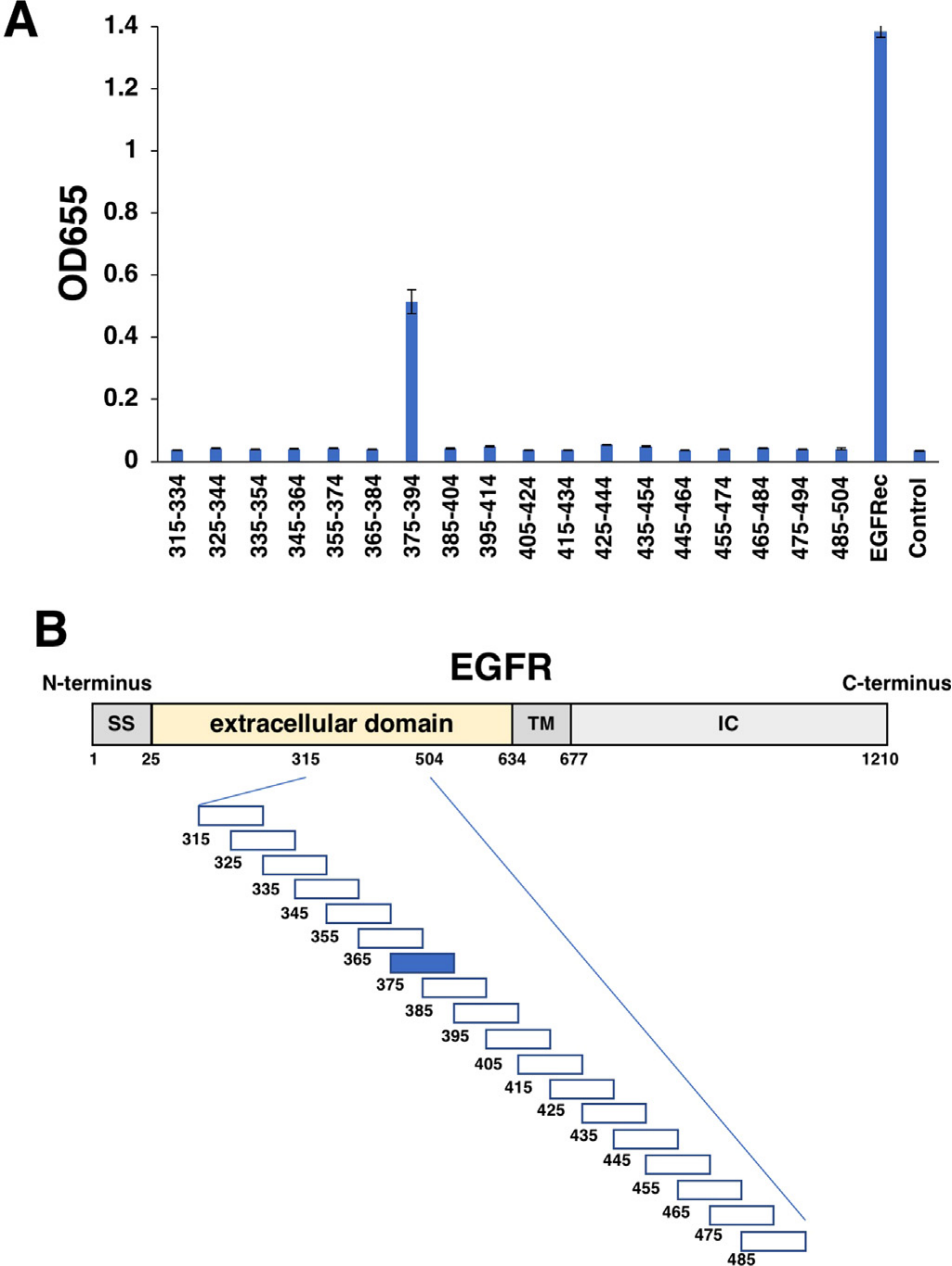
(A) ELISA with EGFR peptides. (B) Schematic illustration of the EMab-134-epitope. EGFRec, EGFR extracellular domain; SS, signal peptide; TM, transmembrane; IC, intracellular.

We next synthesized a series of EGFR peptides (Supplementary Table 1). EMab-134 reacted with the 375–394 aa (_375-_AFRGDSFTHTPPLDPQELDI_−394_) sequence among the peptides and with the extracellular domain of EGFR (EGFRec) on ELISA (Fig. 1 and Fig. 1B).

**Fig. 2:**
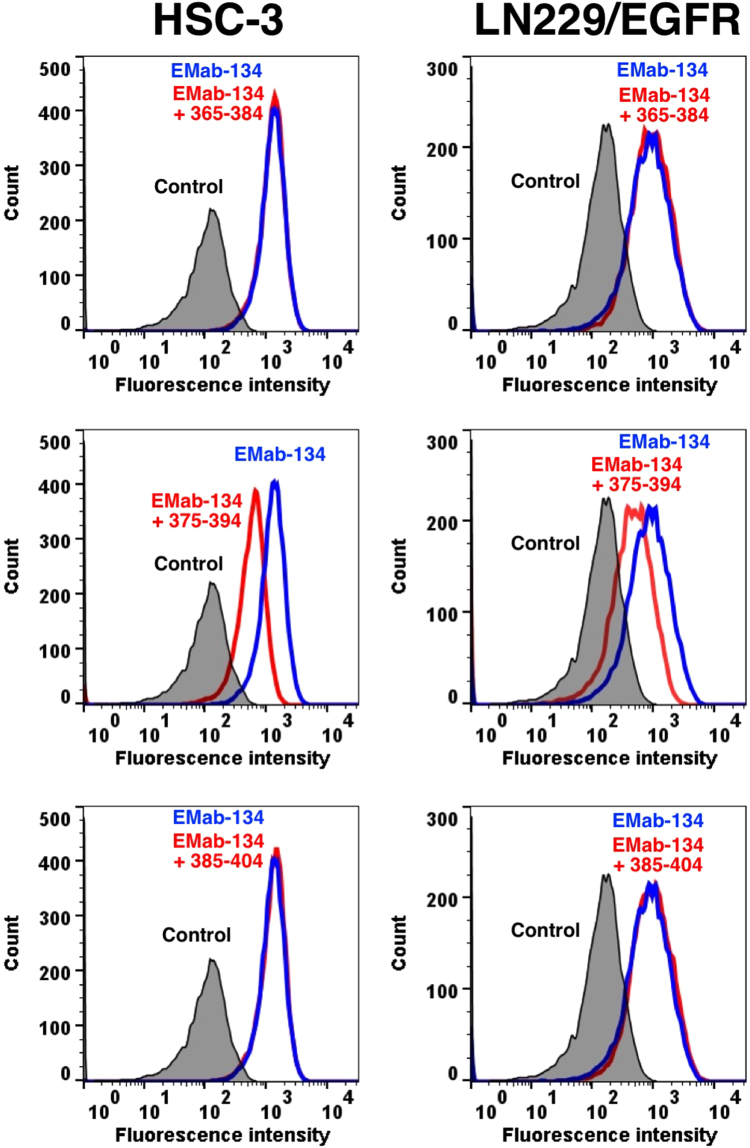
Flow cytometry of HSC-3 and LN229/EGFR cells. The cells were reacted with EMab-134 (10 μg/ml) plus peptides (10 μg/ml) for 30 min at 4 °C, followed by treatment with Alexa Fluor 488-conjugated anti-mouse IgG. Fluorescence data were acquired using the cell analyzer SA3800. Gray peak, negative control; blue peak, EMab-134; red peak, EMab-134 plus each peptide.

We performed a blocking assay using flow cytometry. EMab-134 reacted with the HSC-3 and LN229/EGFR cell lines (Fig. 2). This reaction was partially neutralized by a 375–394-aa sequence. In contrast, the 365–384-aa (_365-_ISGDLHILPVAFRGDSFTHT_−384_) and 385–404-aa (_385-_PPLDPQELDILKTVKEITGF_−404_) sequences did not block the reaction of EMab-134 with both HSC-3 and LN229/EGFR, thereby confirming that the 375–394-aa sequence is a critical epitope of EMab-134. We compared the binding affinity of EMab-134 against a 375–394-aa peptide with that against EGFRec in ELISA. As a result, the binding affinity against EGFRec (*K*_D_: 1.1 × 10^−9^) is much higher than that against a 375–394-aa peptide (*K*_D_: 1.6 × 10^−7^). Therefore, a 375–394-aa peptide might not neutralize EMab-134 reaction completely against HSC-3 and LN229/EGFR cell lines in flow cytometry.

**Fig. 3:**
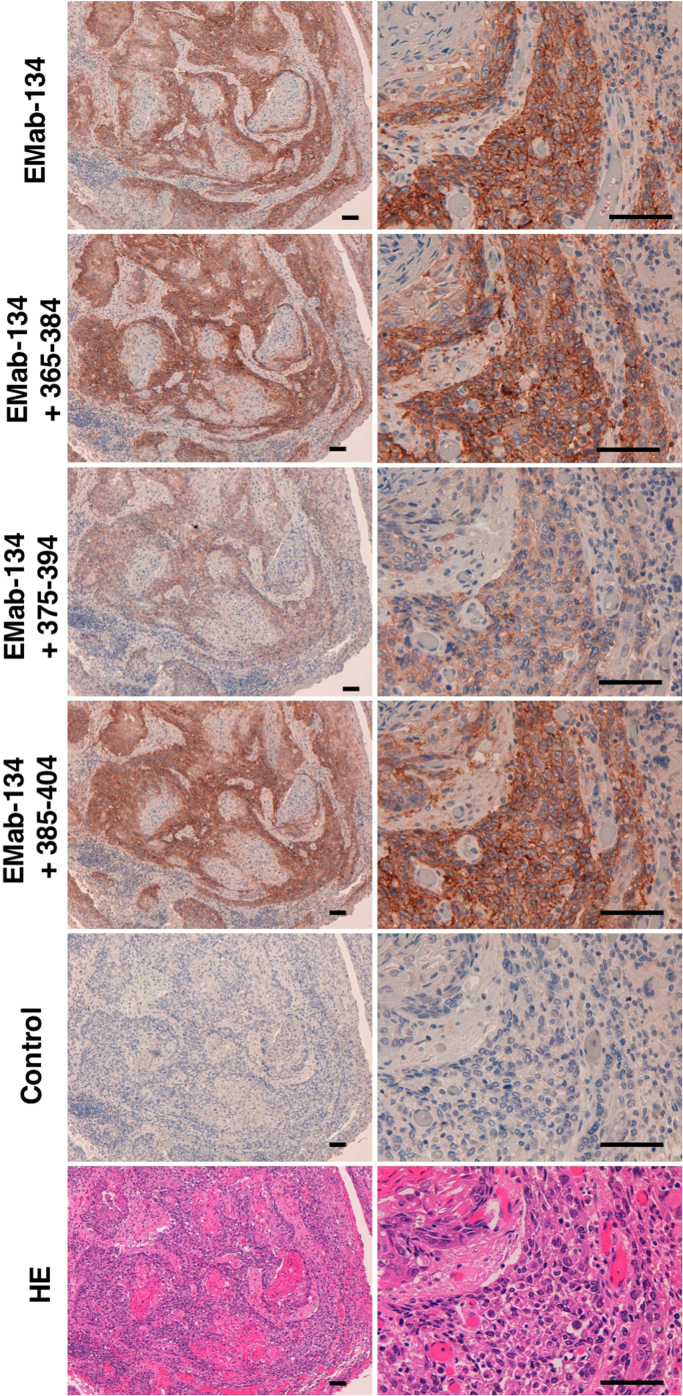
Immunohistochemistry for oral cancers. The sections were incubated with EMab-134 (5 μg/ml) or EMab-134 (5 μg/ml) plus peptides (5 μg/ml) and treated using the Envision^+^ kit. Color development was obtained using 3,3-diaminobenzidine tetrahydrochloride. The sections were then counterstained with hematoxylin. Scale bar = 100 µm.

We next performed a blocking assay using immunohistochemistry and found that EMab-134 reacted with oral cancer tissues (Fig. 3) and that the reaction was neutralized by the 375–394-aa sequence. The 365–384-aa and 385–404-aa sequences did not block the reaction of EMab-134, thereby confirming the results of flow cytometric blocking assays.

**Fig. 4:**
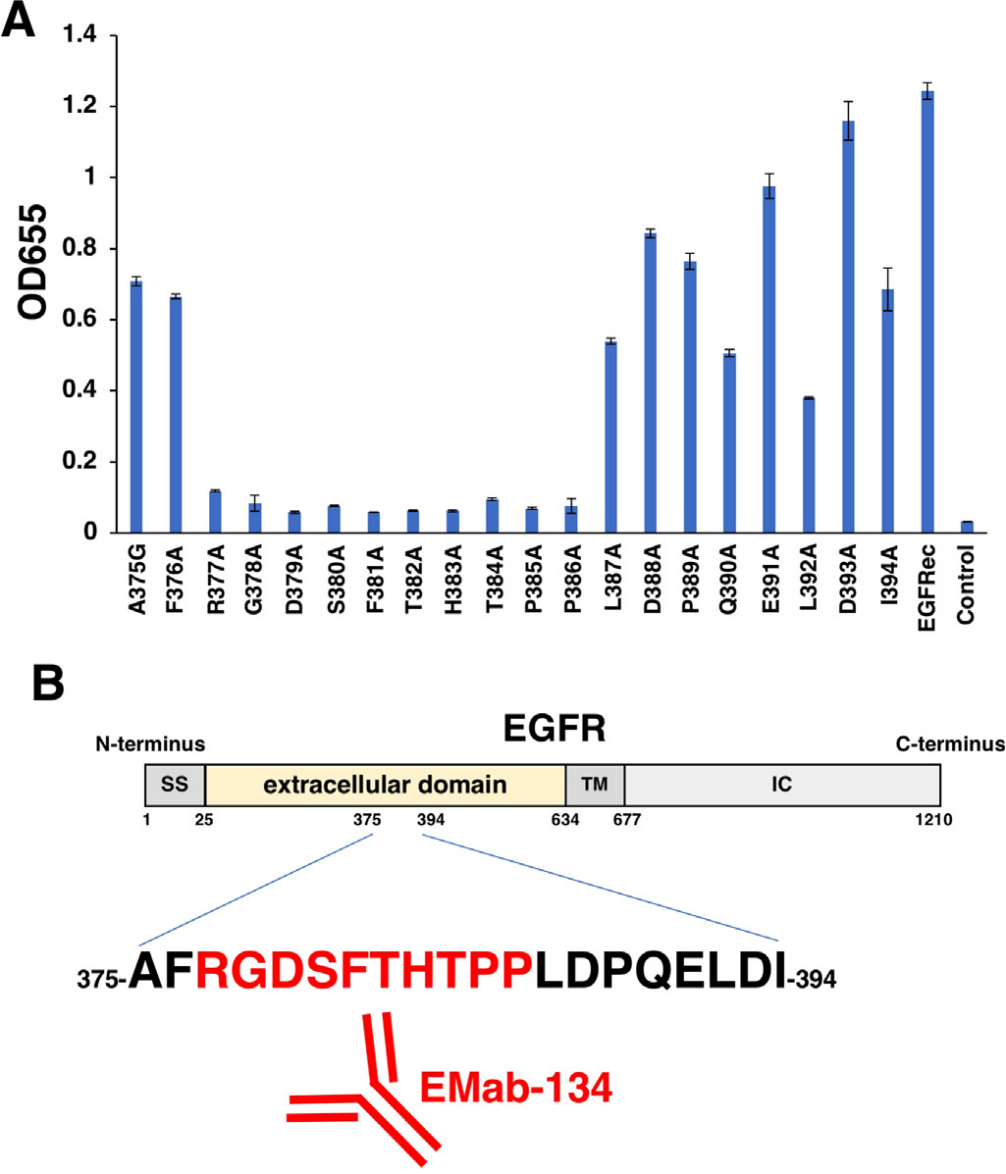
(A) ELISA with EGFR peptides. (B) Schematic illustration of EGFR and the EMab-134-epitope. The critical epitope of EMab-134 is the _377-_RGDSFTHTPP_−386_ sequence. SS, signal peptide; TM, transmembrane; IC, intracellular.

**Fig. S1:**
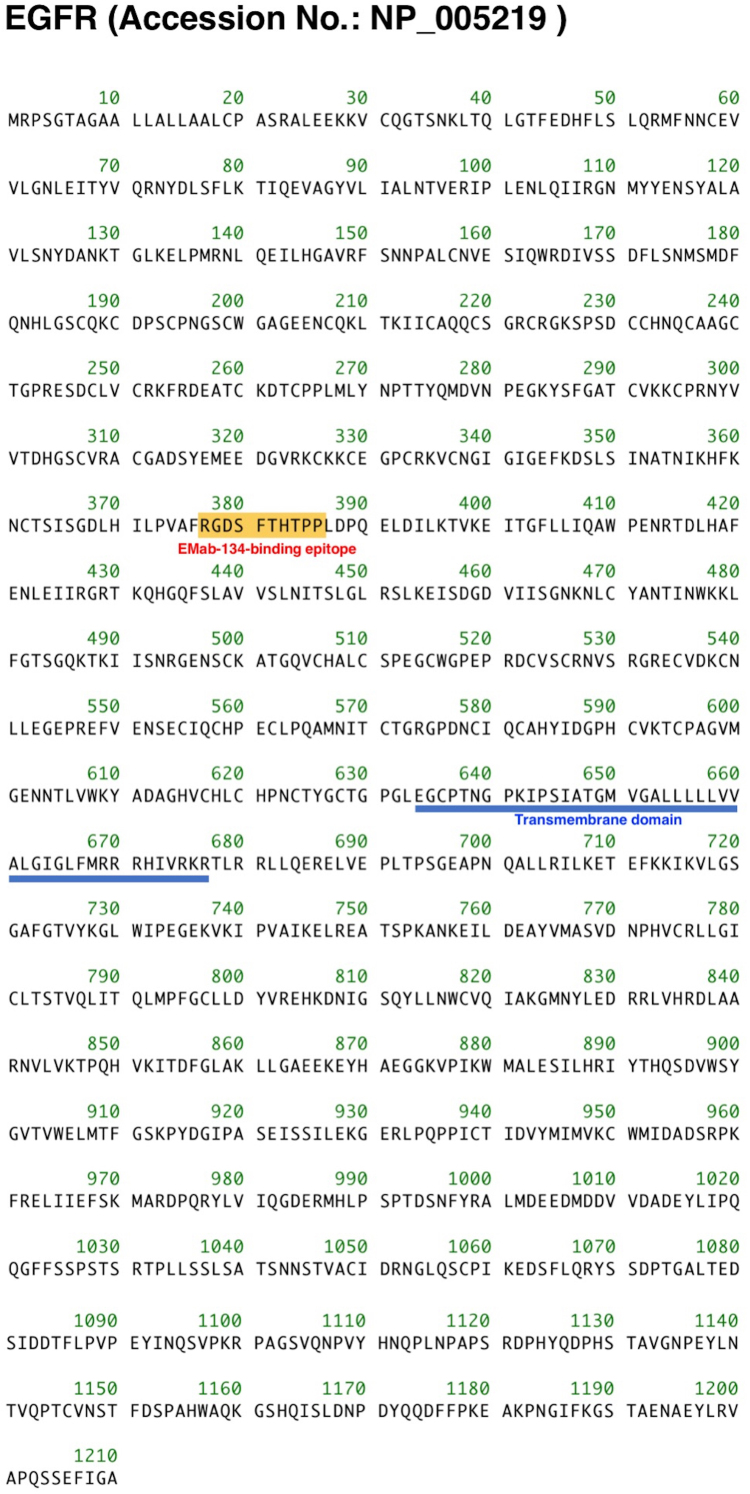
Amino acid sequence of EGFR.

**Fig. S2:**
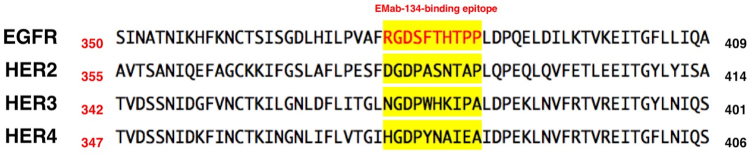
Comparison of acid sequence among HER families.

Finally, we synthesized a series of point mutations of EGFR peptides of length 375–394 aa (Supplementary Table 1). EMab-134 reacted with A375G, F376A, L387A, D388A, P389A, Q390A, E391A, L392A, D393A, and I394A, but not with R377A, G378A, D379A, S380A, F381A, T382A, H383A, T384A, P385A, and P386A, indicating that the EGFR peptides from aa 377 to 386 (_377-_RGDSFTHTPP_−386_) constituted the critical minimum epitope of EMab-134 (Fig. 4 and Supplementary Fig. 1). Although we checked whether the RGDSFTHTPP sequence is conserved in HER family, the identical sequence was not observed in HER2, HER3, and HER4 (Supplementary Fig. 2).

In conclusion, our results indicated that the critical epitope of EMab-134 is the _377-_RGDSFTHTPP_−386_ sequence. Our findings can be used for the production of more functional anti-EGFR mAbs, which would be advantageous for eliciting antitumor effects against EGFR-expressing cancers.
