# Supplementary material for: Elucidation of the critical epitope of an anti-EGFR monoclonal antibody EMab-134
Source: Biochem Biophys Rep. 2018 Apr 12;14:54–7. doi: 10.1016/j.bbrep.2018.03.010 (PMC5986659; doi:10.1016/j.bbrep.2018.03.010)
Supplement: Supplementary file 1 — Supplementary material [file mmc1.docx]

Supplementary table 1. Determination of EMab-134 Epitope by ELISA.

| *Peptide* | *Sequence* | *EMab-134* |
| --- | --- | --- |
| 315-334 | SYEMEEDGVRKCKKCEGPCR | - |
| 325-344 | KCKKCEGPCRKVCNGIGIGE | - |
| 335-354 | KVCNGIGIGEFKDSLSINAT | - |
| 345-364 | FKDSLSINATNIKHFKNCTS | - |
| 355-374 | NIKHFKNCTSISGDLHILPV | - |
| 365-384 | ISGDLHILPVAFRGDSFTHT | - |
| 375-394 | AFRGDSFTHTPPLDPQELDI | ++ |
| 385-404 | PPLDPQELDILKTVKEITGF | - |
| 395-414 | LKTVKEITGFLLIQAWPENR | - |
| 405-424 | LLIQAWPENRTDLHAFENLE | - |
| 415-434 | TDLHAFENLEIIRGRTKQHG | - |
| 425-444 | IIRGRTKQHGQFSLAVVSLN | - |
| 435-454 | QFSLAVVSLNITSLGLRSLK | - |
| 445-464 | ITSLGLRSLKEISDGDVIIS | - |
| 455-474 | EISDGDVIISGNKNLCYANT | - |
| 465-484 | GNKNLCYANTINWKKLFGTS | - |
| 475-494 | INWKKLFGTSGQKTKIISNR | - |
| 485-504 | GQKTKIISNRGENSCKATGQ | - |
| A375G | GFRGDSFTHTPPLDPQELDI | +++ |
| F376A | AARGDSFTHTPPLDPQELDI | ++ |
| R377A | AFAGDSFTHTPPLDPQELDI | - |
| G378A | AFRADSFTHTPPLDPQELDI | - |
| D379A | AFRGASFTHTPPLDPQELDI | - |
| S380A | AFRGDAFTHTPPLDPQELDI | - |
| F381A | AFRGDSATHTPPLDPQELDI | - |
| T382A | AFRGDSFAHTPPLDPQELDI | - |
| H383A | AFRGDSFTATPPLDPQELDI | - |
| T384A | AFRGDSFTHAPPLDPQELDI | - |
| P385A | AFRGDSFTHTAPLDPQELDI | - |
| P386A | AFRGDSFTHTPALDPQELDI | - |
| L387A | AFRGDSFTHTPPADPQELDI | ++ |
| D388A | AFRGDSFTHTPPLAPQELDI | +++ |
| P389A | AFRGDSFTHTPPLDAQELDI | +++ |
| Q390A | AFRGDSFTHTPPLDPAELDI | ++ |
| E391A | AFRGDSFTHTPPLDPQALDI | +++ |
| L392A | AFRGDSFTHTPPLDPQEADI | + |
| D393A | AFRGDSFTHTPPLDPQELAI | +++ |
| I394A | AFRGDSFTHTPPLDPQELDA | ++ |

＋＋＋, OD655≧0.7; ＋＋, 0.5 ≦OD655＜0.7;＋, 0.2≦OD655＜0.5; —, OD655＜0.2.
